# Differentiating poor prognosis from treatment-related fluctuations in Guillain-Barré syndrome for repeating immunoglobulin treatment: an illustrative case report

**DOI:** 10.3389/fimmu.2026.1889220

**Published:** 2026-09-03

**Authors:** Alessio D’Elia, Luciana Mascia, Luca Gregorio Giaccari, Angela Comanducci, Barbara Maistrello, Emilio Lozupone, Monica Santo Sabato, Mariangela Bianco, Guglielmo Lucchese

**Affiliations:** 1 Neurology Unit, “Vito Fazzi” Hospital, Lecce, Italy; 2 Department of Experimental Medicine, University of Salento, Lecce, Italy; 3 Anesthesiology and Intensive Care Unit, “Vito Fazzi” Hospital, Lecce, Italy; 4 IRCCS Fondazione Don Carlo Gnocchi, Milan, Italy; 5 Neuroradiology Unit, “Vito Fazzi” Hospital, Lecce, Italy

**Keywords:** CMV (cytomegalovirus), GBS, IVIG, re-treatment, TRF

## Abstract

**Background:**

Guillain-Barré syndrome (GBS) is an acute immune-mediated polyradiculoneuropathy that may follow infectious triggers and can progress to severe weakness, cranial nerve involvement, autonomic dysfunction, and respiratory failure. Cytomegalovirus (CMV)-associated GBS is often linked to a more severe clinical course. Although intravenous immunoglobulin (IVIG) and plasma exchange are established first-line treatments, management becomes challenging when patients deteriorate after initial therapy, particularly when distinguishing poor prognosis from treatment-related fluctuation (TRF).

**Case presentation:**

We report the case of a 42-year-old woman who developed progressive paresthesias, diffuse pain, gait disturbance, areflexia, facial involvement, dysphagia, dysarthria, autonomic dysfunction, and later respiratory failure. Cerebrospinal fluid analysis showed albuminocytologic dissociation, electrodiagnostic studies supported acute polyradiculoneuropathy, and MRI demonstrated contrast enhancement of the facial nerve roots and cauda equina. Serum testing revealed CMV IgM positivity with detectable CMV DNA, consistent with CMV-associated GBS. The patient received IVIG and ganciclovir. After initial stabilization following the first IVIG course, she experienced sudden respiratory deterioration requiring intubation. Given the temporal pattern of stabilization followed by worsening, the episode was interpreted as TRF occurring in a patient with otherwise poor prognostic features. A second IVIG cycle was administered, followed by gradual respiratory and neurological improvement.

**Discussion:**

This case illustrates the clinical difficulty of deciding whether repeated immunotherapy is justified in severe GBS. Current evidence discourages routine second IVIG courses in patients with poor prognosis who fail to respond; however, this recommendation does not necessarily apply to TRF, where renewed immunotherapy may be considered. In this patient, post-IVIG stabilization followed by acute deterioration supported the interpretation of TRF; persistent CMV viremia was considered a possible marker of ongoing immune stimulation.

**Conclusion:**

CMV-associated GBS may follow a severe and fluctuating course. Careful distinction between poor prognosis, nonresponse, and treatment-related fluctuation is essential because it directly influences therapeutic decision-making. Close monitoring, individualized immunotherapy, intensive supportive care, and early rehabilitation remain central to optimizing outcomes in severe GBS.

## Introduction

Guillain-Barré syndrome (GBS) is an acute, immune-mediated polyradiculoneuropathy that represents the most common cause of acute flaccid paralysis worldwide (1). It is typically characterized by rapidly progressive, symmetric weakness of the limbs, often accompanied by sensory disturbances and diminished or absent deep tendon reflexes. Autonomic dysfunction, cranial nerve involvement, and respiratory compromise may occur in severe cases, making early recognition and management critical (2).

GBS is usually preceded by an infectious event, most commonly respiratory or gastrointestinal, which triggers an aberrant immune response leading to demyelination and/or axonal damage (3). Among infectious triggers, cytomegalovirus (CMV) is recognized as a significant cause, frequently associated with a more severe clinical course, including cranial neuropathies and autonomic involvement (4).

The two established disease-modifying treatments for GBS are intravenous immunoglobulin (IVIG) and plasma exchange, both showing comparable effectiveness (5). Nevertheless, some patients experience fluctuating or relapsing courses, which can complicate treatment and prognosis. Reporting and analyzing such atypical or severe presentations is essential to enhance understanding of the disease spectrum, optimize management strategies, and improve patient outcomes (6).

## Case presentation

A 42-year-old woman presented to our emergency department on 3 August 2025 with palpitations, holocranial headache, and paresthesias in the upper limbs. On admission, she was found to have high blood pressure. Neurological examination, cranial CT, intracranial CT angiography, and transcranial Doppler were unremarkable, and she was discharged with advice for follow-up at a headache clinic.

Ambulatory ECG monitoring revealed sinus tachycardia, and beta-blocker therapy was initiated; blood pressure was normal. In the following days, she developed persistent cervical pain, diffuse arthromyalgia, nausea, and loss of appetite. Rheumatologic evaluation suggested fibromyalgia, and duloxetine therapy was started but soon discontinued due to worsening gastrointestinal symptoms.

**Figure 1:**
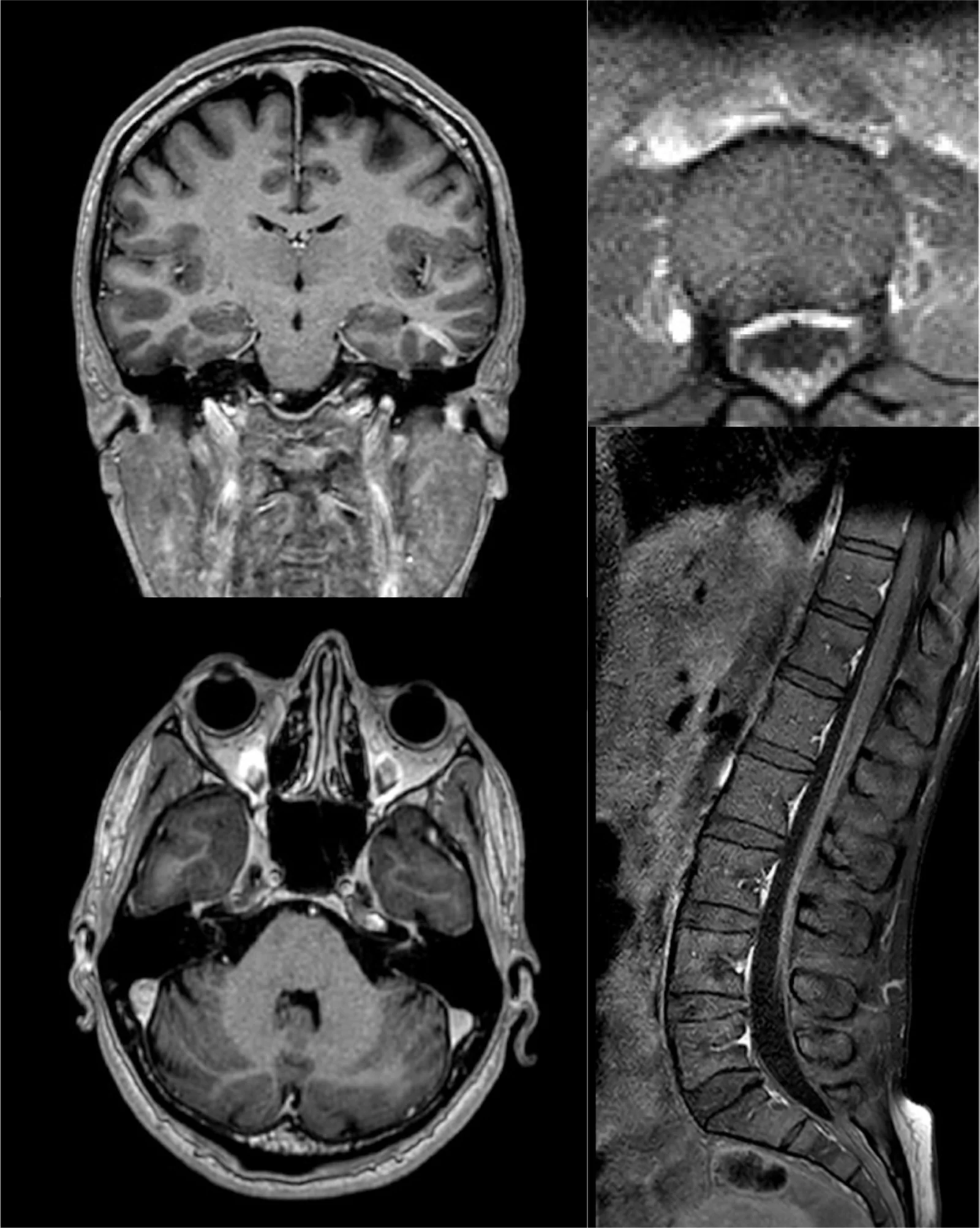
Contrast-enhanced MRI findings. Gadolinium-enhanced T1-weighted brain images in the coronal and axial planes show bilateral enhancement of the intracranial facial nerves; gadolinium-enhanced T1-weighted lumbar spine images in the axial and sagittal planes show enhancement of the cauda equina nerve roots.

**Figure 2:**
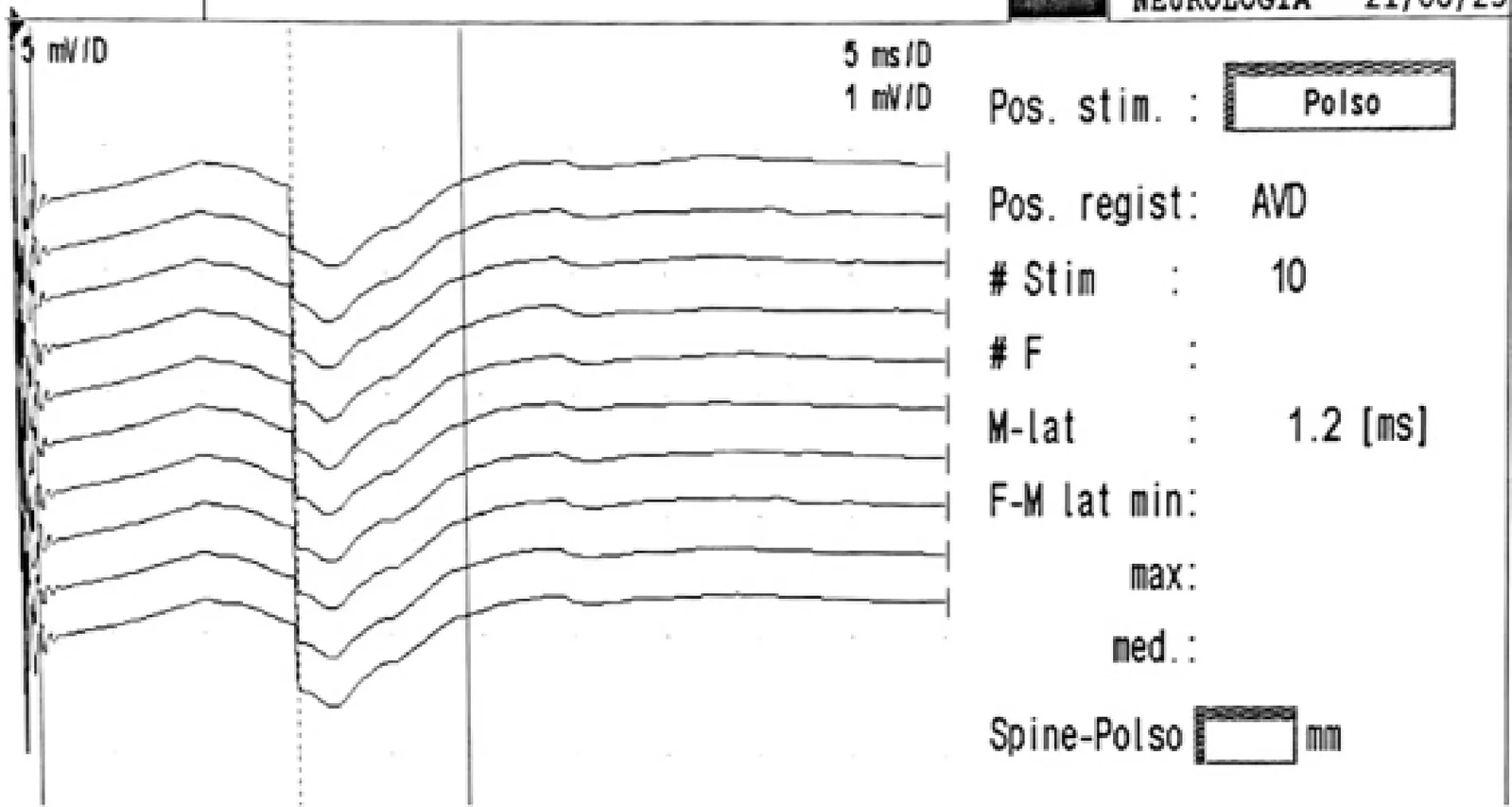
Representative left ulnar nerve F-wave tracing at the 10-day follow-up, showing absent F-wave responses.

Nine days after initial presentation and discharge from the emergency department, the patient was admitted to our neurology unit because of a worsening clinical picture—including diffuse muscle pain, limb dysesthesias (predominantly in the lower limbs), recurrent vomiting, anosmia, and ageusia. Neurological examination revealed facial muscle spasms, gait disturbance, and absent deep tendon reflexes in the lower limbs. Laboratory tests showed elevated liver enzymes with negative serology for viral hepatitis and increased creatine kinase (CK) levels. Although exact numerical values were not available for retrospective extraction, hyperCKemia has been described in GBS and may reflect muscle injury secondary to acute motor denervation rather than a primary myopathic process (7, 8). Brain and spinal MRI showed contrast enhancement of the intracranial segments of the facial nerves bilaterally and of the roots of the cauda equina (**Figure 1**). Electrodiagnostic studies demonstrated early signs of acute polyradiculoneuropathy (**Figure 2**; **Table 1**). Cerebrospinal fluid (CSF) analysis revealed elevated proteins with normal cell count, supporting the diagnosis of Guillain-Barré syndrome (GBS) (**Table 2**). CSF testing for autoimmune encephalitis antibodies and common infectious agents was negative. The patient was started on intravenous immunoglobulin (IVIG, 0.4 g/kg/day for 5 days).

**Table 1:** Serial nerve conduction study findings at baseline (t0), day 10 follow-up, and 6-month follow-up, including motor and sensory response amplitudes, conduction velocities, and latencies.

Amplitude (mV)	t0	day 10 f-u	6 months f-u
Motor nerves			
L Medianus			
Wrist – APB	4.1	0.2	/
Elbow – wrist	3.6	0.1	/
L Ulnaris			
Wrist – AVD	8.2	0.2	9.9
Dis. Elbow - wrist	8.2	0.4	8.8
R Tibial Nerve			
Med. Mal – Abd. Hall.	6.7	0.3	0.4
Popl. Fossa – Med.mall.	5.4	0.3	0.8
L Tibial Nerve			
Med. Mall. – Abd. Hall	6.5	0.3	0.4
Popl. Fossa – Med.mall	5.9	0.2	0.7
L Peroneus			
Ankle – Ext. Brev. Digit	1.6	/	/
Dis. C.F. – ankle	1.3	/	/
R Peroneus			
Ankle – Ext. Brev. Digit	1.4	/	/
Dis. C.F. – ankle	1.2	/	/
Sensory nerves			
L Suralis			
Calf – Lat. Mall.	n.a.	10.0	4.4
Lat. Mall – Calf	n.a.	10.0	3.0
R Suralis			
Calf – Lat. Mall.	n.a.	8.9	2.8

Conduction velocity (m/s)	t0	day 10 f-u	6 months f-u
Motor nerves			
L Medianus			
Elbow – wrist	50.0	/	/
L Ulnaris			
Elbow – wrist	54.7	41.1	46.8
R Tibial Nerve			
Popl. Fossa -Med.mall	40.8	42.2	14.0
L Tibial Nerve			
Popl. Fossa -Med.mall	40.0	39.1	18.1
L Peroneus			
Dis. C.F. - ankle	36.9	/	/
R Peroneus			
Dis. C.F. - ankle	42.8	/	/
Sensory nerves			
L Suralis			
Calf – Lat. Mall	/	38.1	32.4
R Suralis			
Calf – Lat. Mall	/	/	26.8
Latencies (msec)	t0	day 10 f-u	6 months f-u
Motor nerves			
L Medianus			
Wrist – APB	6.5	12.5	n.a.
Elbow – wrist	11.2	18.3	n.a
L Ulnaris			
Wrist – AVD	2.5	5.7	3.8
Dist. Elbow - wrist	7.8	11.3	8.5
R Tibial Nerve			
Med. Mal – Abd. Hall.	4.5	9.7	13.2
Popl. Fossa – Med.mall.	13.2	18.0	38.2
L Tibial Nerve			
Med. Mall. – Abd. Hall	3.8	6.5	15.8
Popl. Fossa – Med.mall	13.3	15.7	34.0
Sensory nerves			
L Suralis			
Calf – Lat. Mall.	/	2.1	3.7
R Suralis			
Calf – Lat. Mall.	/	2.1	4.1

**Table 2:** Cerebrospinal fluid findings at diagnostic lumbar puncture, demonstrating albuminocytologic dissociation with elevated protein and albumin levels and a normal white blood cell count.

CSF parameter	Result	Reference range
Aspect	Clear, colorless	
RBC	absent	
CSF/blood gluc. Ratio (%)	58.7	>45
glucose (g/L)	0.54	0.40–0.70
proteins (mg/dL)	104.9*	14–45
albumin (mg/dL)	59.5*	13.9–24.6
albumin index (%)	18.03*	<7
WBC (cells/uL)	3	0–5

Serum screening for neurotropic viruses revealed positivity for CMV IgM, with confirmation of viral DNA in blood (3,198 IU/mL), thus suggesting parainfectious GBS. CMV-PCR testing of CSF was negative. Therefore, antiviral therapy with ganciclovir (5 mg/kg twice daily) was initiated. Accordingly, three weeks after the initiation of antiviral therapy, CMV DNA was no longer detectable in the blood, and IgG seroconversion was documented. The thrombophilia and autoimmune serum panel was negative except for anti-smooth muscle antibody (ASMA) at a titer of 1:80. Moreover, a total-body CT scan was performed, which showed no lesions suggestive of neoplastic processes.

During the first three days of IVIG treatment, the neurological status rapidly deteriorated, with the onset of marked tetraparesis (predominantly in the lower limbs), dysphagia, severe facial diplegia with lagophthalmos, dysarthria, and voiding difficulties requiring urinary catheterization. In accordance with the electrophysiological findings, no objective sensory deficits were observed. Autonomic dysfunction was also evident, characterized by persistent sinus tachycardia, for which beta-blocker therapy was required. Cognitive-behavioral and sleep disturbances (anxiety, agitation, hallucinations, and insomnia) emerged, and symptomatic treatment with mirtazapine and antipsychotics was attempted, though with limited benefit. Hyponatremia was also detected, requiring intravenous hypertonic fluid therapy. A second brain MRI showed no evidence of CNS involvement, and the cognitive-behavioral symptoms were interpreted as a multifactorial delirium in the context of severe GBS, with hyponatremia, sleep disruption, critical illness, and pharmacological treatment, including a possible contribution from ganciclovir (9), considered as potential contributing factors.

Five days after completion of the IVIG cycle, following an initial period of stabilization, the patient suddenly experienced further clinical deterioration, with dyspnea and evidence of ventilatory respiratory failure, requiring endotracheal intubation and transfer to the intensive care unit. A right pneumothorax with basal hypoventilation complicated jugular central line placement, but resolved rapidly. Modified Erasmus GBS Outcome Score (mEGOS) at day 7, which remained unchanged at the time of worsening with respiratory failure, was 10, indicating poor prognosis.

The first extubation attempt, four days after intubation, was unsuccessful because of persisting respiratory muscle weakness. Despite the high mEGOS, a second 5-day IVIG cycle was started because the respiratory worsening following initial stabilization after the first treatment was judged to be a treatment-related fluctuation (TRF). Twelve days after intubation, a gradual improvement in the overall clinical and neurological condition was observed. Maximal inspiratory pressure (MIP) increased from approximately 12 to 25, allowing for a successful extubation with no need for tracheostomy, and for resumption of spontaneous breathing under regular respiratory physiotherapy. During the stay in the intensive care unit, a urinary tract infection caused by *Klebsiella pneumoniae* was identified and treated with targeted antibiotic therapy.

After readmission to our neurology ward, the patient continued to show rapid improvement, also supported by ongoing physiotherapy and swallowing rehabilitation. She regained the ability to perform postural changes and to feed herself orally. Communication skills had also markedly improved due to the resolution of dysarthria and recovery of facial muscle strength. Mild tetraparesis and gait unsteadiness persisted, partly related to muscle mass loss, which had nonetheless been limited by an appropriate nutritional plan. Once full clinical and hemodynamic stability was achieved, the patient was discharged and transferred to a specialized facility for intensive neurorehabilitation.

At the 120-day follow-up, neurological examination revealed residual left facial nerve palsy, mild weakness in the upper limbs, and more pronounced weakness in the lower limbs, predominantly affecting the distal muscles, where impaired deep sensation was also noted. Deep tendon reflexes were absent. The patient was able to walk short distances with assistance and a wide-based gait. Compared with the previous electrodiagnostic assessment, findings were interpreted as consistent with a recovering sensorimotor polyradiculoneuropathy, predominantly demyelinating. Electrophysiological findings also suggested secondary axonal involvement of the facial nerve, more pronounced on the left side. Brain MRI demonstrated persistent contrast enhancement along the course of the facial nerves (from the internal auditory canal to the exit at the petrous bone) and of the trigeminal nerves, bilaterally.

## Discussion

We describe a 42-year-old woman with severe CMV-associated Guillain-Barré syndrome (GBS), characterized by initial rapid progression, cranial neuropathies, and autonomic dysfunction. Diagnosis was supported by absent deep tendon reflexes, albuminocytologic dissociation, electrophysiological evidence of acute polyradiculoneuropathy, and MRI showing nerve root enhancement. Comprehensive management included antiviral therapy for CMV, intensive supportive care, and vigilant monitoring of autonomic and metabolic complications.

A distinctive feature of this case was the neurological course in relation to immunotherapy, characterized by rapid deterioration during the initial IVIG course, subsequent clinical stabilization, and later abrupt respiratory worsening. The patient nevertheless had a poor predicted outcome, with an mEGOS of 10 at day 7. Further therapeutic possibilities were evaluated by a multidisciplinary team. Three options were available: first, therapeutic plasma exchange (TPE) following the initial IVIG cycle; second, an additional IVIG cycle; third, further supportive therapy alone.

The following considerations guided the decision. Current guidelines for treating GBS do not recommend combining IVIG and TPE in any sequential order due to a lack of evidence of additional benefit (10, 11). Moreover, TPE nine days after completion of the first IVIG cycle might wash out the therapeutic immunoglobulins—an approach that would be expensive and irrational (12–14). Furthermore, CMV DNA remained detectable during the unsuccessful weaning attempt, indicating ongoing infection. TPE has been used in viral infections, mainly for removing cytokines, but the procedure might also remove critically important neutralizing antibodies (15, 16).

Not only is a second cycle of IVIG not recommended, but current guidelines even advise against it in nonresponding GBS cases with poor prognosis, mainly in light of recent data showing an association with worse outcomes with repeated immunoglobulin treatment in such patients (10, 17). However, these recommendations do not necessarily apply to treatment-related fluctuations (TRF), in which renewed deterioration occurs after an initial response or stabilization following treatment (10, 11, 18). In such cases, repeating the initial therapy may be considered, based on the hypothesis that treatment produced a transient beneficial effect while the underlying autoimmune process remained active.

In our patient, neurological deterioration during the initial IVIG course was followed, after treatment completion, by a five-day period without further progression before the sudden development of respiratory failure. Although this interval does not fulfill the >1-week stabilization criterion included in the original operational definition of TRF (19), subsequent clinical literature has adopted a broader clinical concept of TRF, encompassing renewed neurological deterioration after an initial period of improvement or stabilization (11). Notably, Ramakrishnan et al. classified patients as having TRF on the basis of new neurological deterioration after an initial recovery or plateau phase, even when the formal Kleyweg criteria were not fulfilled (20). Accordingly, the clinical course observed in our patient was considered compatible with TRF.

In light of these considerations, we interpreted the sudden respiratory deterioration following post-IVIG stabilization as a TRF and opted for a second IVIG cycle. A successful weaning process, good recovery, and positive neurorehabilitative course followed.

## Conclusions

CMV-associated GBS may present with severe deficits and treatment-related fluctuations (TRF). The present case report does not provide causal evidence for repeated IVIG in TRF; rather, it illustrates the clinical reasoning underlying retreatment in a patient whose course was considered compatible with TRF. Moreover, the clinical presentation highlights the importance of considering a short but clearly documented period of clinical stabilization, as opposed to uninterrupted progression, when evaluating the possibility of TRF and subsequent therapeutic options. It is important to stratify patients with poor prognosis on the basis of the presence or absence of TRF because, in patients with TRF, retreatment with the initial therapy may be considered, although supporting evidence remains limited (10, 11). Switching therapy from IVIG to TPE, or vice versa, remains unrecommended owing to a lack of evidence of any benefit. Close monitoring and timely adaptation of immunotherapy are critical. Multidisciplinary supportive care and rehabilitation remain essential for recovery.

## Data Availability

The original contributions presented in the study are included in the article/supplementary material. Further inquiries can be directed to the corresponding author.
